# Supplementary material for: Shape memory polymer resonators as highly sensitive uncooled infrared detectors
Source: Nat Commun. 2019 Oct 4;10:4518. doi: 10.1038/s41467-019-12550-6 (PMC6778134; doi:10.1038/s41467-019-12550-6)
Supplement: Supplementary file 1 — Supplementary Information [file 41467_2019_12550_MOESM1_ESM.docx]

**Shape Memory Polymer Resonators as Highly Sensitive Uncooled Infrared Detectors**

Ulas Adiyan^[[1]](#footnote-1)^, Tom Larsen^2^, Juan José Zárate^1^, Luis Guillermo Villanueva^2^, Herbert Shea^1^

**SUPPLEMENTARY FIGURES**

*
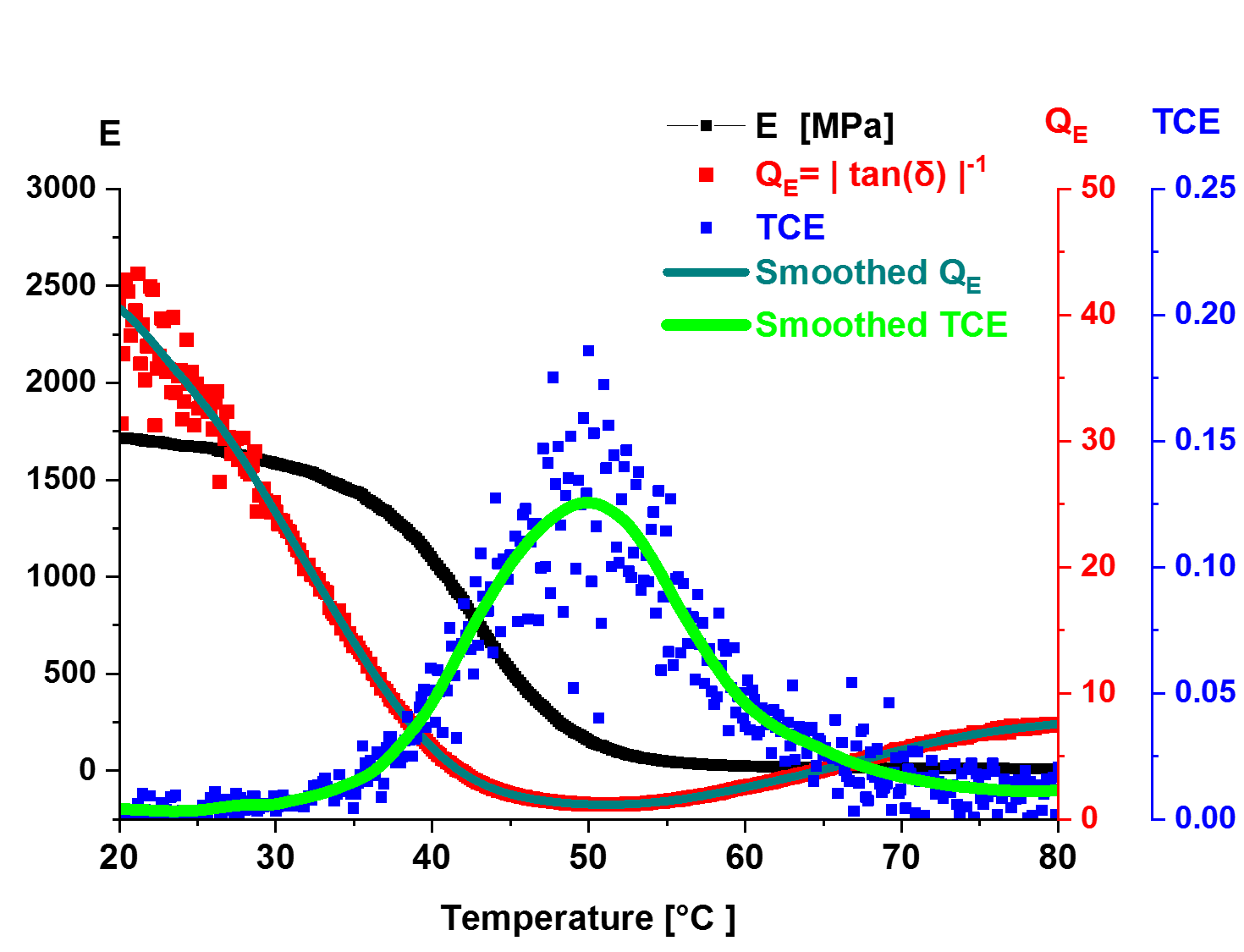
*

**Supplementary Figure 1: Dynamic mechanical analysis (DMA) measurements:** Young’s modulus (E) and intrinsic quality factor ($Q_{E}$) with respect to temperature for the shape memory polymer (SMP) membrane (*MM4520 SMP pellets from SMP Technologies*). $Q_{E}$ is the inverse of the loss factor $tan(\delta)$, $Q_{E}= \left| tan(\delta) \right|^{-1}=E'/E''$. $E'$ is the storage modulus and $E''$ is the loss modulus.$Q_{E}$ measurements show low quality factor around the glass transition temperature. The thermal coefficient of the Young’s Modulus (TCE) with respect to temperature is plotted in blue, which was calculated from Young’s modulus (*E*) data vs temperature measurement. The smoothed versions of the plots correspond to “50 points” LOESS (locally estimated scatterplot smoothing).

*
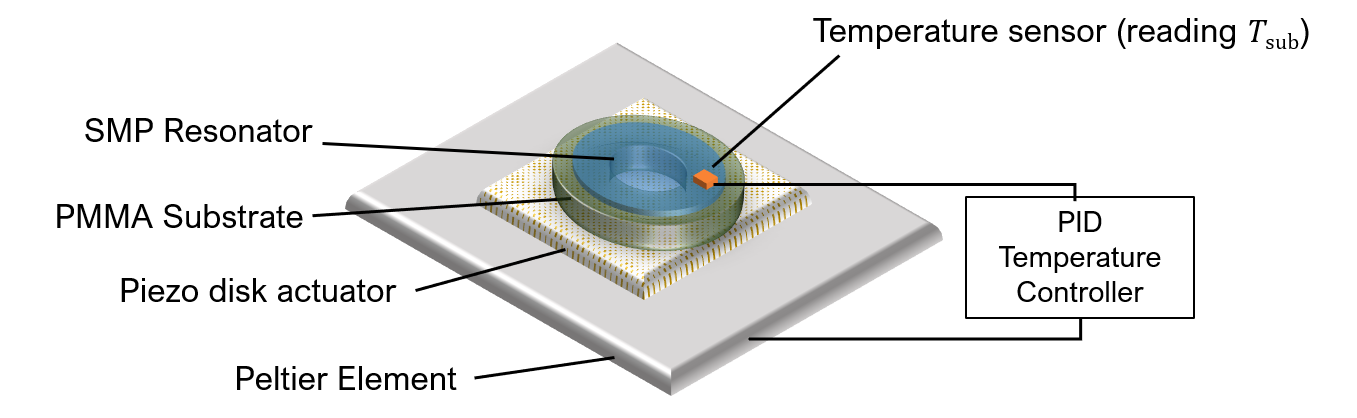
*

**Supplementary Figure 2:** **The placement of the resonator.** The shape memory polymer (SMP) resonator (with a radius of 520 μm and a thickness of 10 μm) on a Polymethyl methacrylate (PMMA) substrate with a thickness of 1mm. The resonator is attached on a piezo disk actuator and placed on a heater with a proportional–integral–derivative (PID) temperature controller system, which controls the operation temperature of the resonator (*T*_sub_), using a temperature sensor placed on the substrate.

*
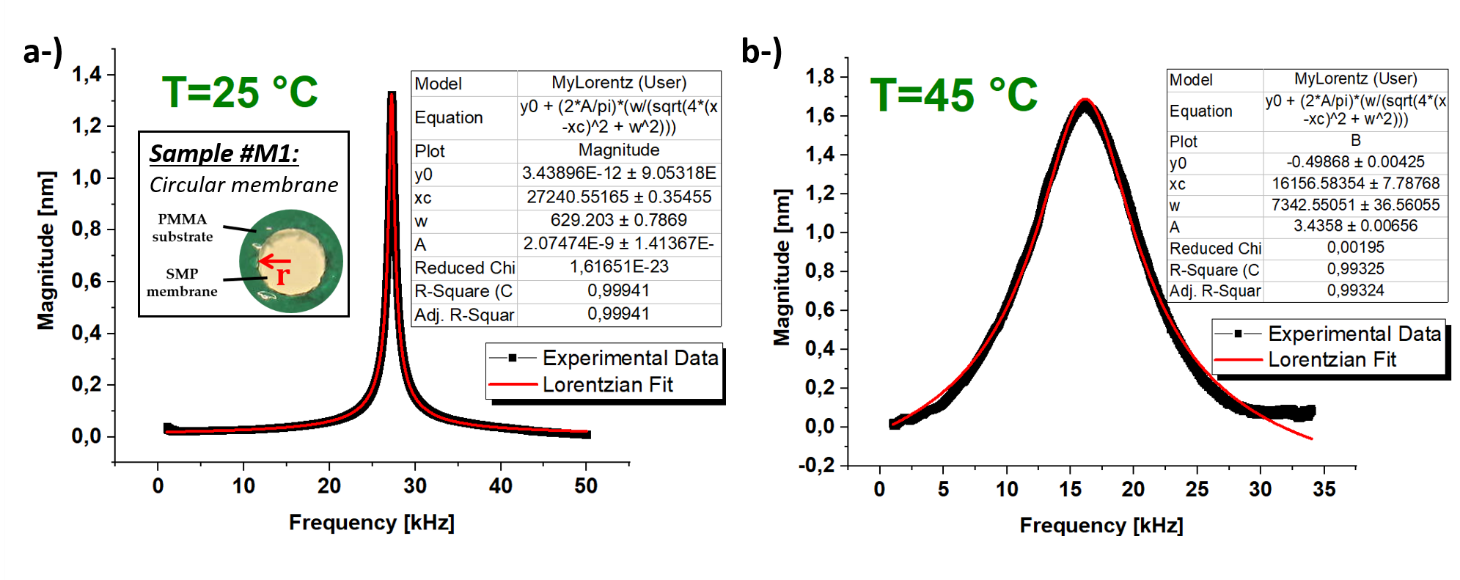
*

**Supplementary Figure 3:** **Displacement vs. frequency of the resonator.** The shape memory polymer (SMP) circular resonator has a radius of 520 μm and thickness of 10 μm, measured by Laser Doppler Vibrometer (LDV), a-) at 25°C and b-) at 45 °C.

***
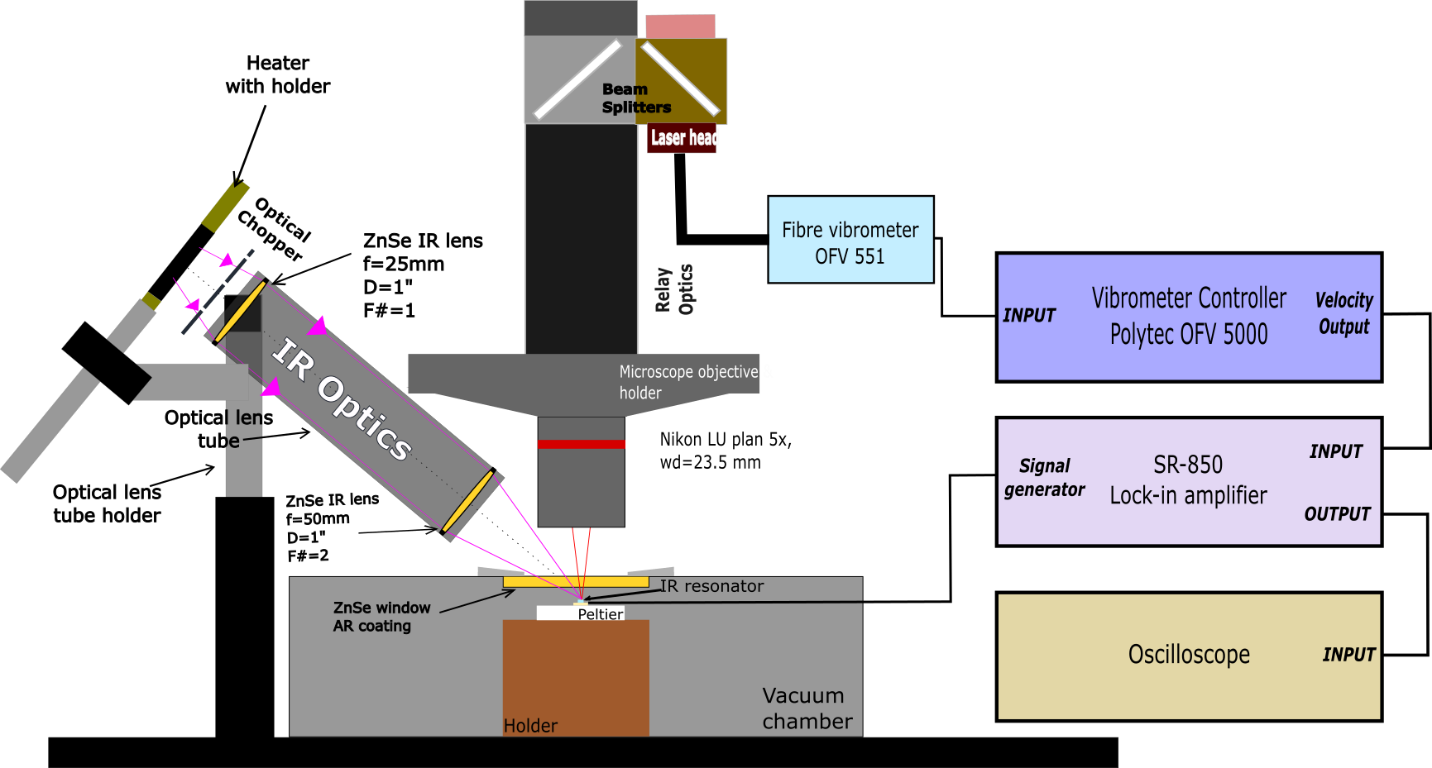
***

**Supplementary Figure 4: Schematic of the experimental setup for the characterization.**  The setup is utilized to detect the resonant frequency shift of the infrared (IR) shape memory polymer (SMP) sensor, which is subjected to IR radiation.

**

**Supplementary Figure 5:** **Absorption spectrum of the shape memory polymer (SMP) membranes.** 10 μm, 23 μm and 57 μm thick SMP sheets are used in the wavelength range of 7 μm – 14 μm. A THERMO Nicolet 8700 Fourier transform infrared (FTIR) spectrometer is used to obtain the infrared (IR) absorption of the SMP with different thicknesses.

*
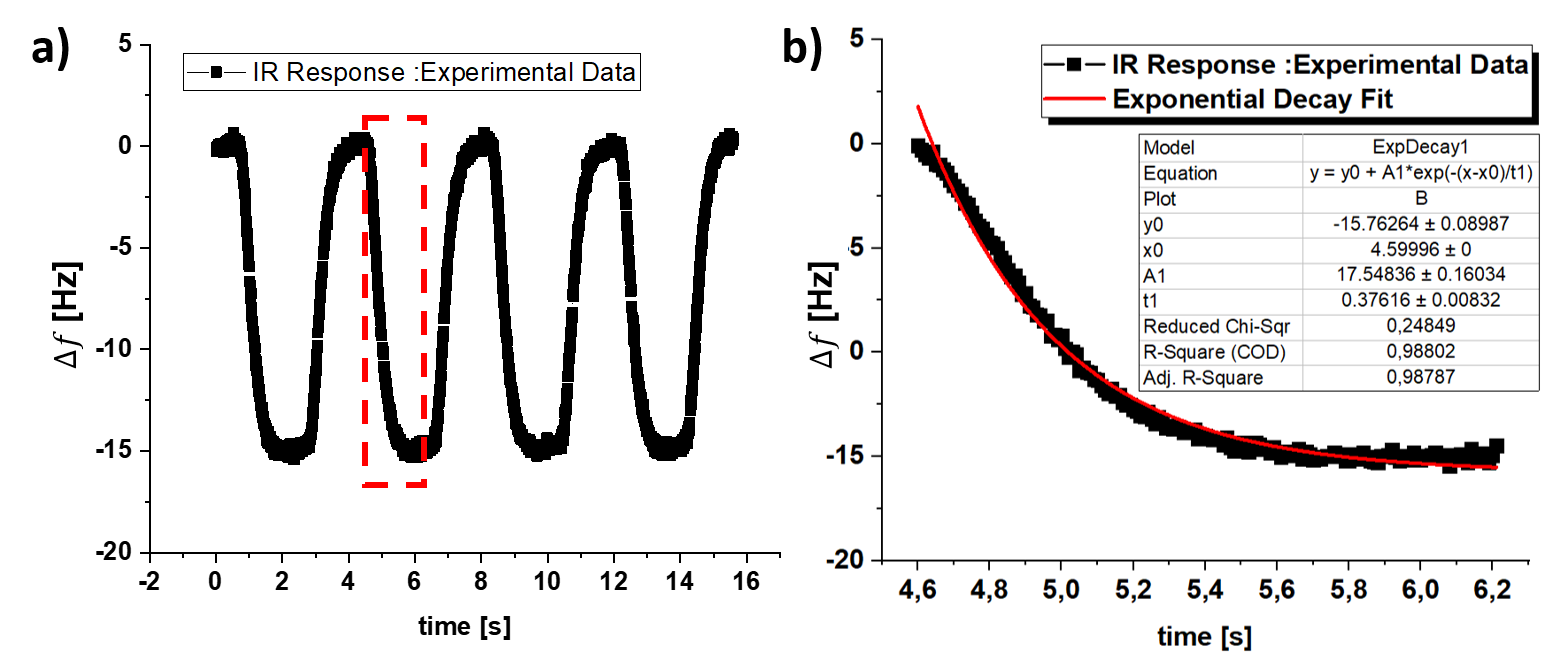
*

**Supplementary Figure 6:** **Thermal time constant measurement. a-)** The frequency response of the infrared (IR) shape memory polymer (SMP) sensor in vacuum at *T*_sub_*=*30 °C operation temperature when the target temperature is periodically changed by *ΔT*_bb_ *=*5°. **b-)** Thermal time constant of 376$\pm$ 8 ms determined from an exponential fit to the IR response data.

**
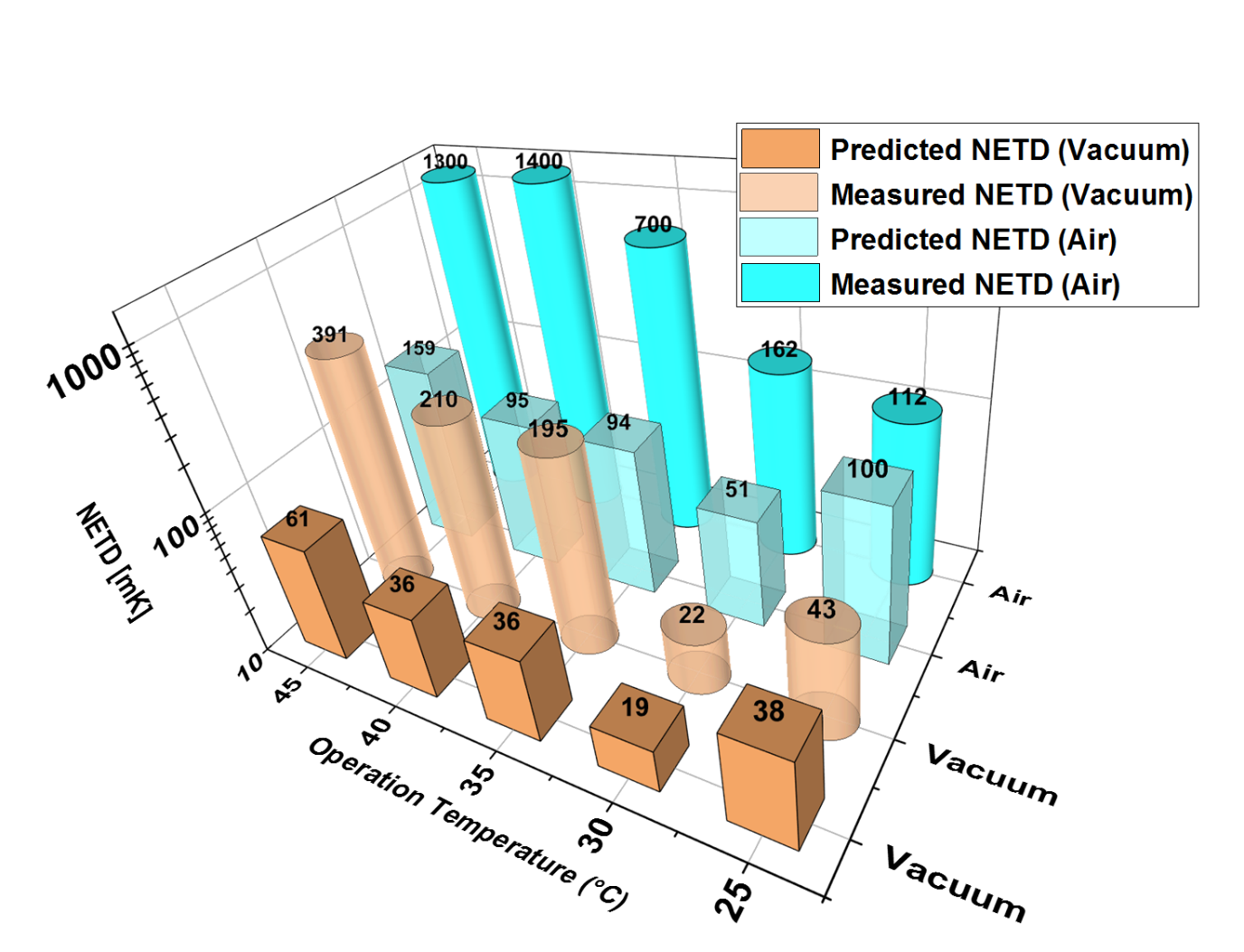
**

**Supplementary Figure 7:** **The measured and predicted noise equivalent temperature difference** **(NETD) vs. operation temperature (***T*_sub_**).** The measured NETDs in vacuum and in air, along with the predicted NETD.

**
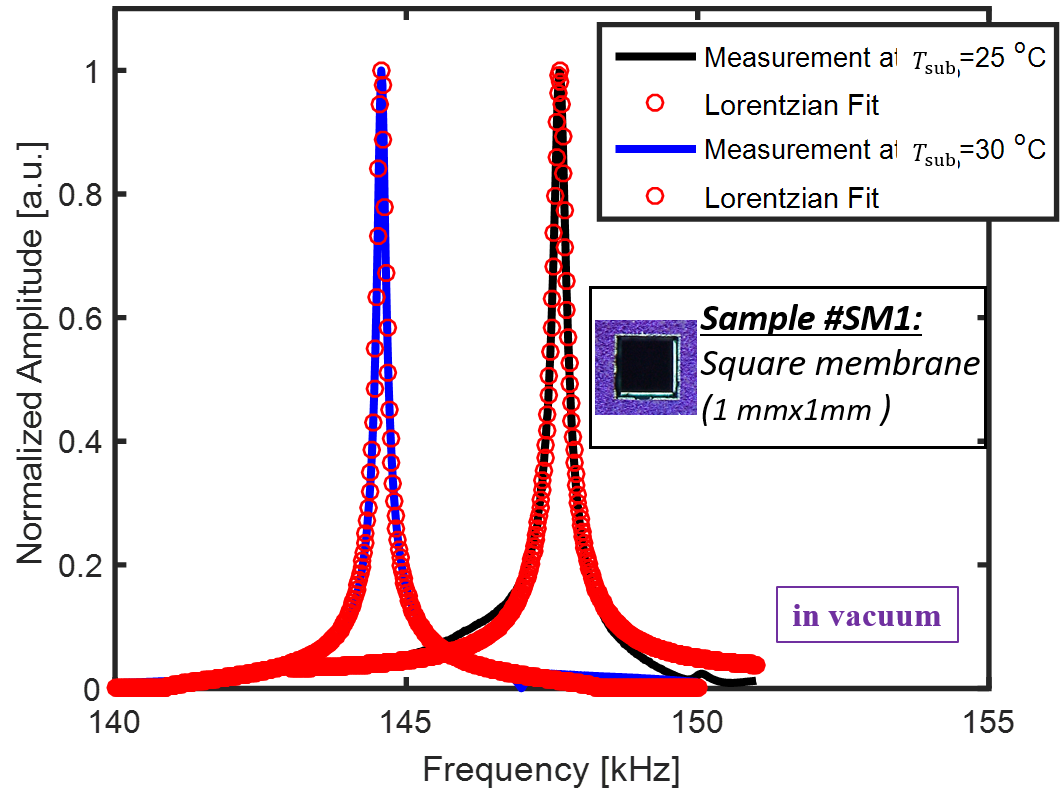
**

**Supplementary Figure 8: Displacement vs. frequency of the bimorph resonators.** SiN_x_ + SMP membrane at 25 °C and at 30 °C is measured by Laser Doppler Vibrometer (LDV). The size of the square membrane is 1 mm x 1 mm and the thickness of the SMP is 15 μm.

**
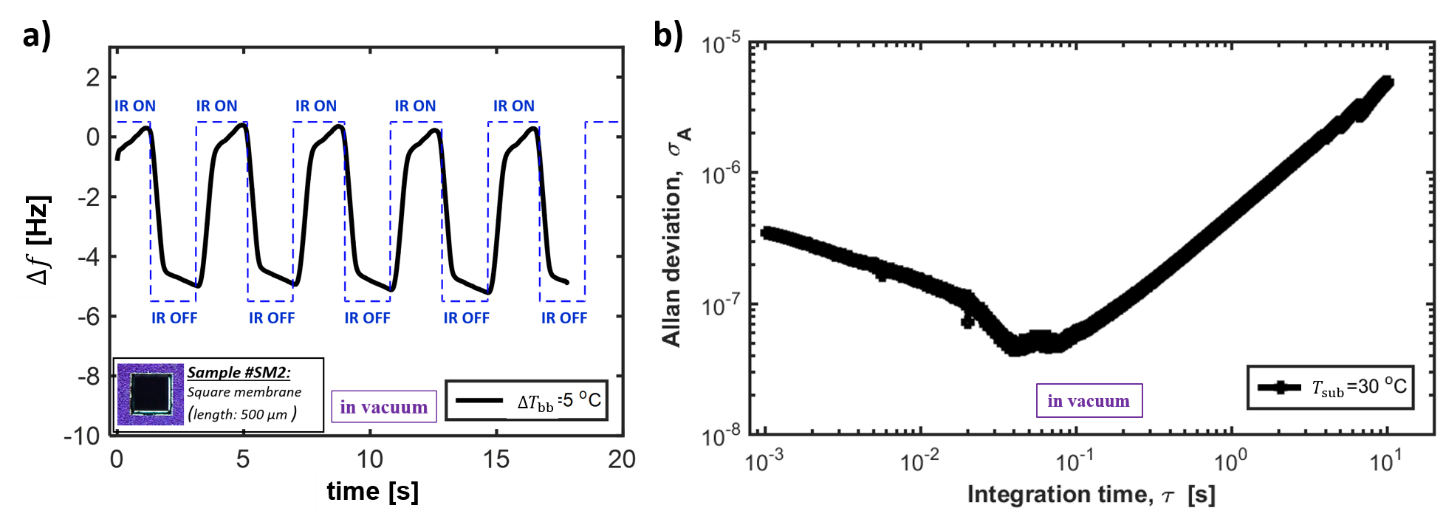
**

**Supplementary Figure 9:** **Noise equivalent temperature difference** (**NETD) measurements for the bimorph resonator.** a-) The frequency response of the smaller SiN_x_ + SMP infrared resonant sensor in vacuum at *T*_sub_*=*30 °C operation temperature when the target temperature is periodically changed by *ΔT*_bb_ *=*5°. A moving average filter (N=101) is used to smooth the data, which was acquired with an acquisition rate of 1.8 kHz. b-) Allan Deviation measurements as a function of integration time at *T*_sub_ *=*30 °C for the same IR resonant sensor in vacuum (~ ${10}^{-3}$ Pa). The top view of the fabricated resonator is shown at the bottom left corner of the figure.


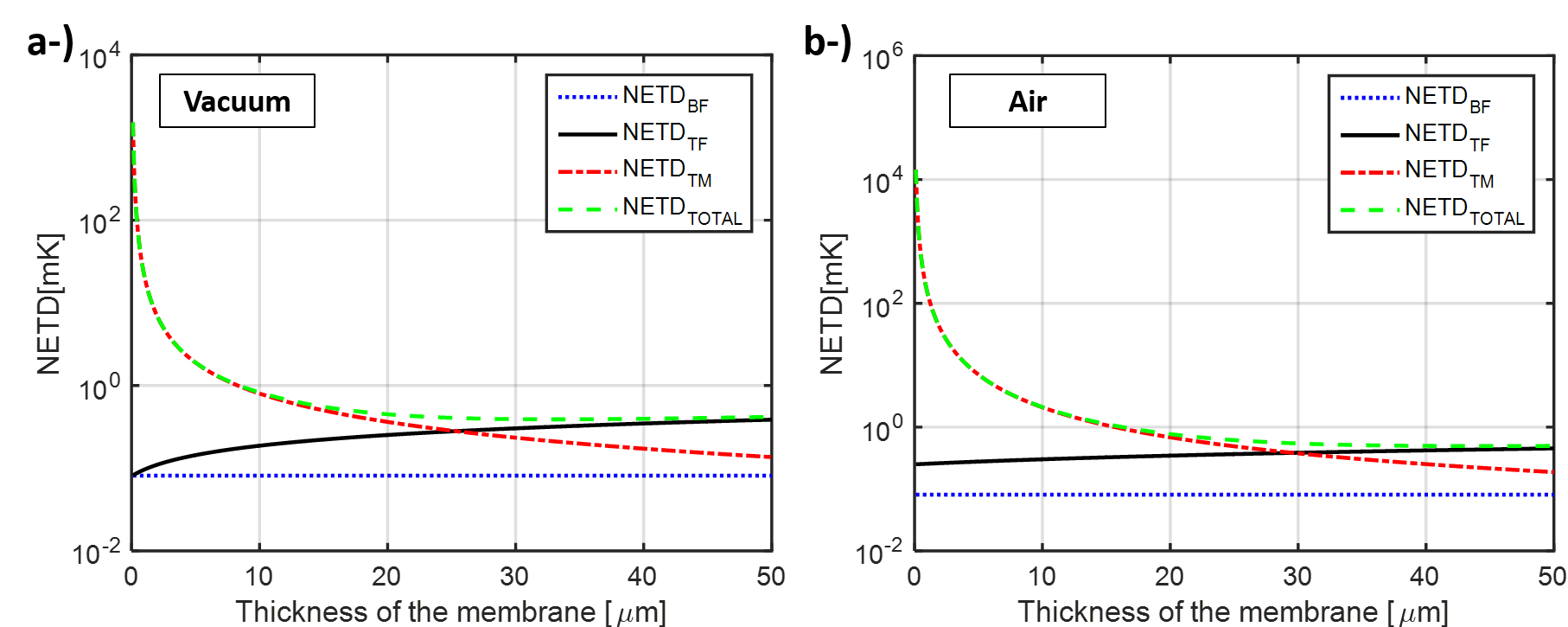


**Supplementary Figure 10:** **Noise equivalent temperature difference** (**NETD) calculation vs. thickness of the SMP membrane.** The calculation is made for a fixed radius (r= 520 µm – which is the radius of Sample #M1) at the operation temperature *T*_sub_ = 30 °C. a-) in vacuum b-) in air. *NETD*_BF_ : Background fluctuation noise, *NETD*_TF_: Thermal fluctuation noise, *NETD*_TM_: Thermomechanical noise.


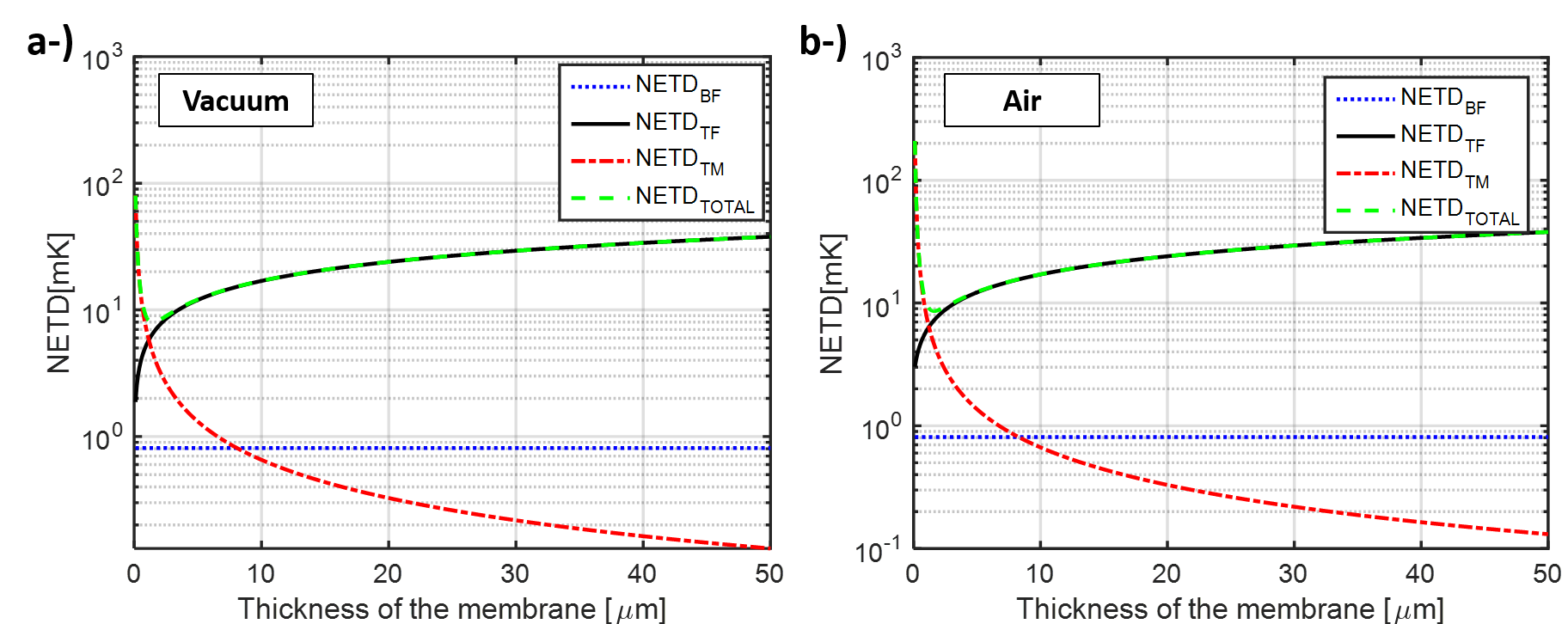


**Supplementary Figure 11:** **Noise equivalent temperature difference** (**NETD) calculation vs. thickness for** **a smaller SMP membrane.** The calculation is made for a fixed radius (r= 52 µm – ie 10 times smaller than in Supplementary Figure 10) at the operation temperature *T*_sub_ = 30 °C. a-) in vacuum b-) in air. *NETD*_BF:_ Background fluctuation noise, *NETD*_TF_: Thermal fluctuation noise, *NETD*_TM_: Thermomechanical noise.


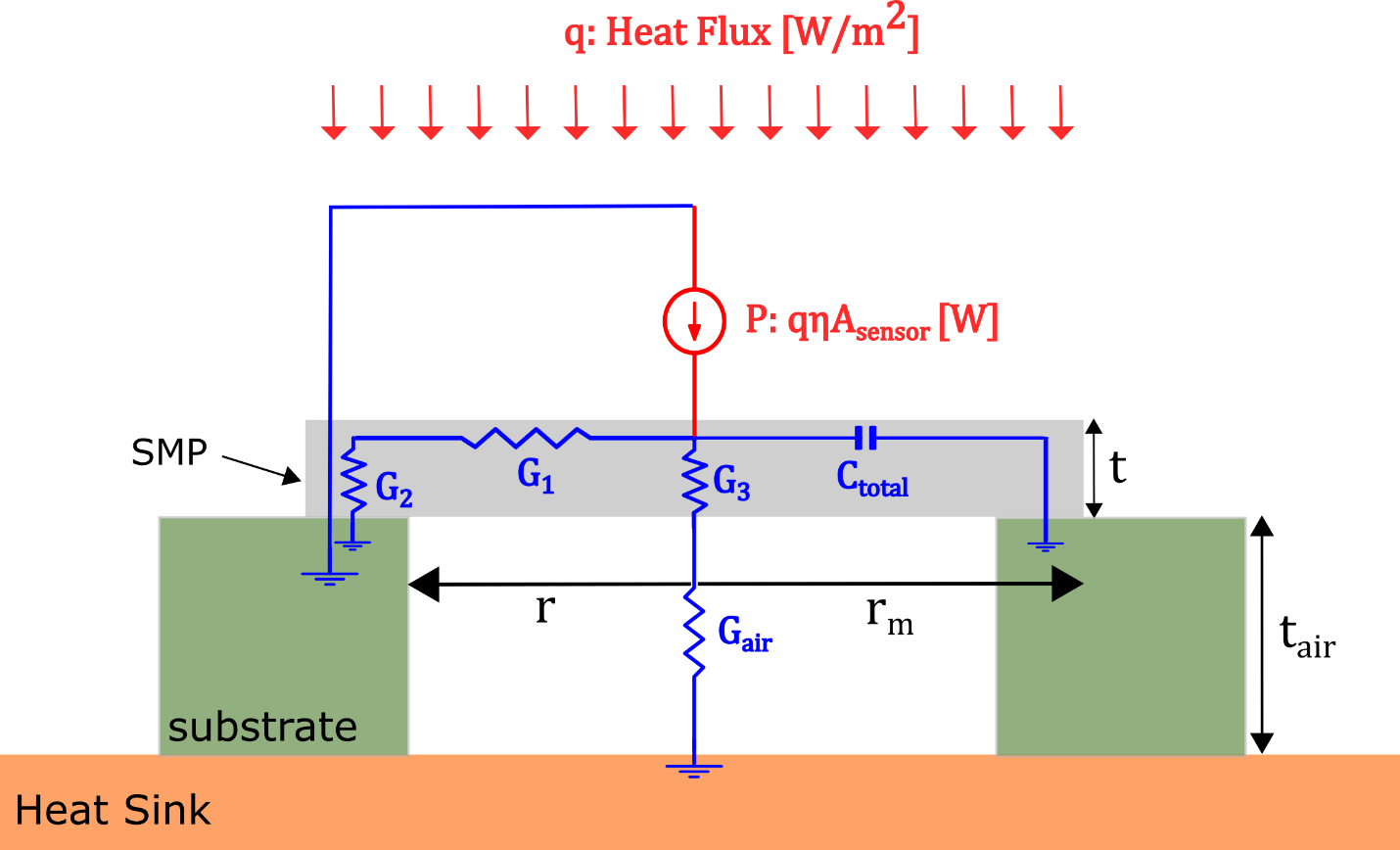


**Supplementary Figure 12: The thermal lumped model for the SMP-based IR sensor.** A heat flux is applied on the membrane and the heat flow from the membrane to the substrate goes through the indicated thermal resistances, which is shown with the equivalent circuit of the thermal model.


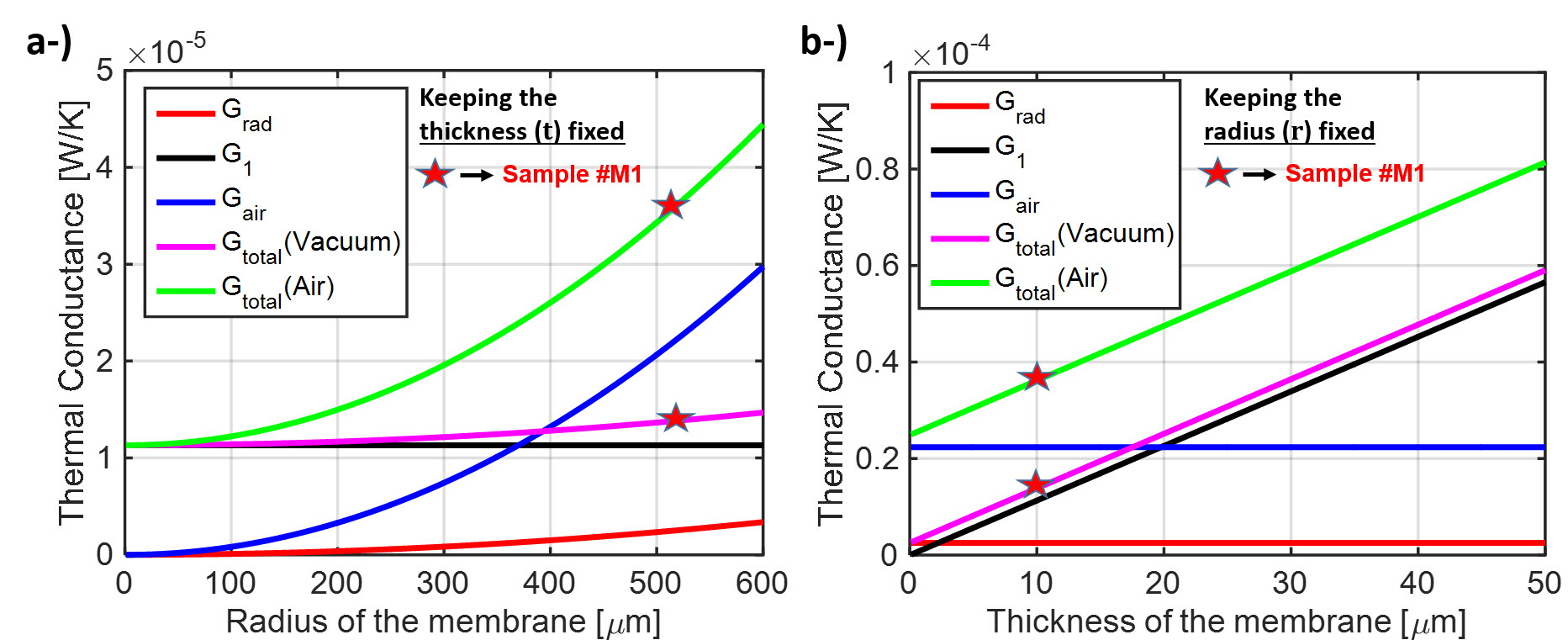


**Supplementary Figure 13*:*** **Thermal conductance analysis of the SMP infrared sensor.** a-) Thermal conductance vs. radius of the membrane of the membrane for a fixed thickness (*t=* 10 µm). b-) Thermal conductance vs thickness of the membrane for a fixed radius (r= 520 µm). The contribution of the conduction through radiation, through air, through the membrane and the total thermal conductance in vacuum and in air were plotted for the operation temperature T = 30 °C. The red star shows the tested sample #M1 on both plots.


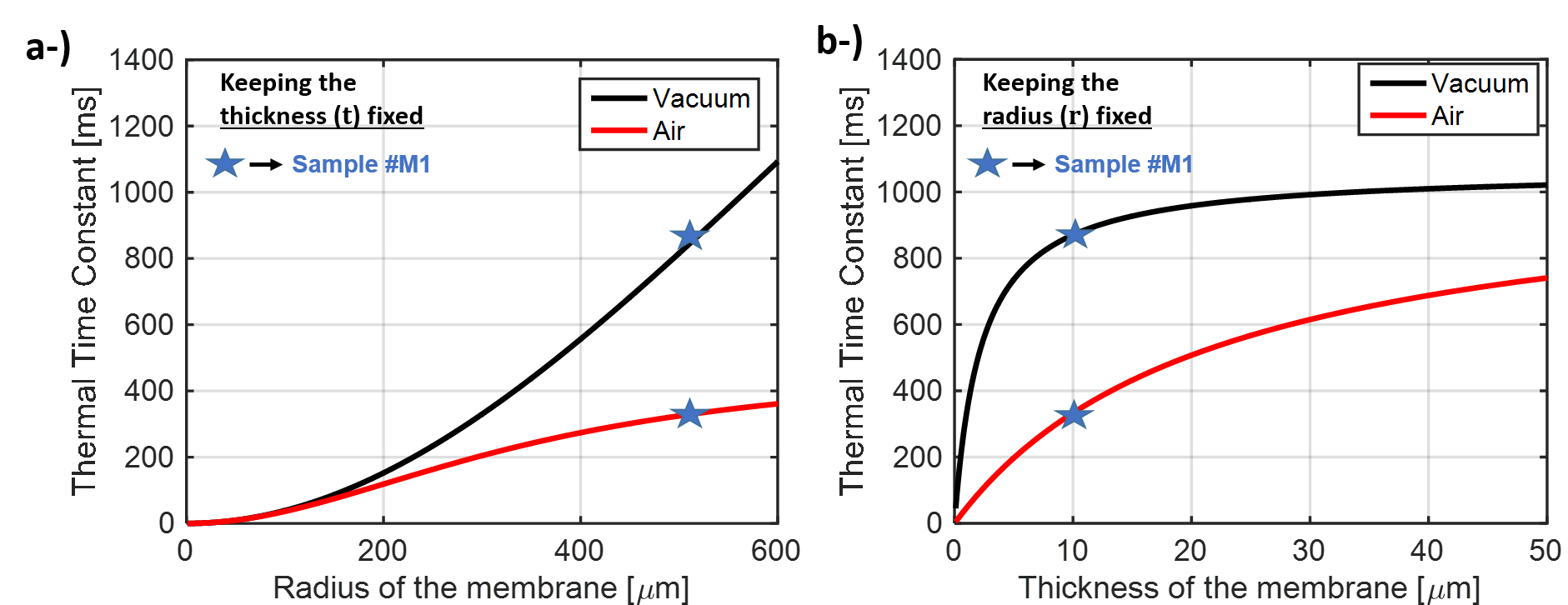


**Supplementary Figure 14*:*** **Thermal time constant analysis of the** **infrared sensor.** a-) Thermal time constant vs radius of the membrane of the membrane for a fixed thickness (*t*= 10 µm). b-) Thermal time constant vs thickness of the membrane for a fixed radius (*r*= 520 µm). The thermal time constant in vacuum and in air were plotted for the operation temperature *T*_sub_ = 30 °C. The blue star shows the tested sample, Sample #M1 on both plots.


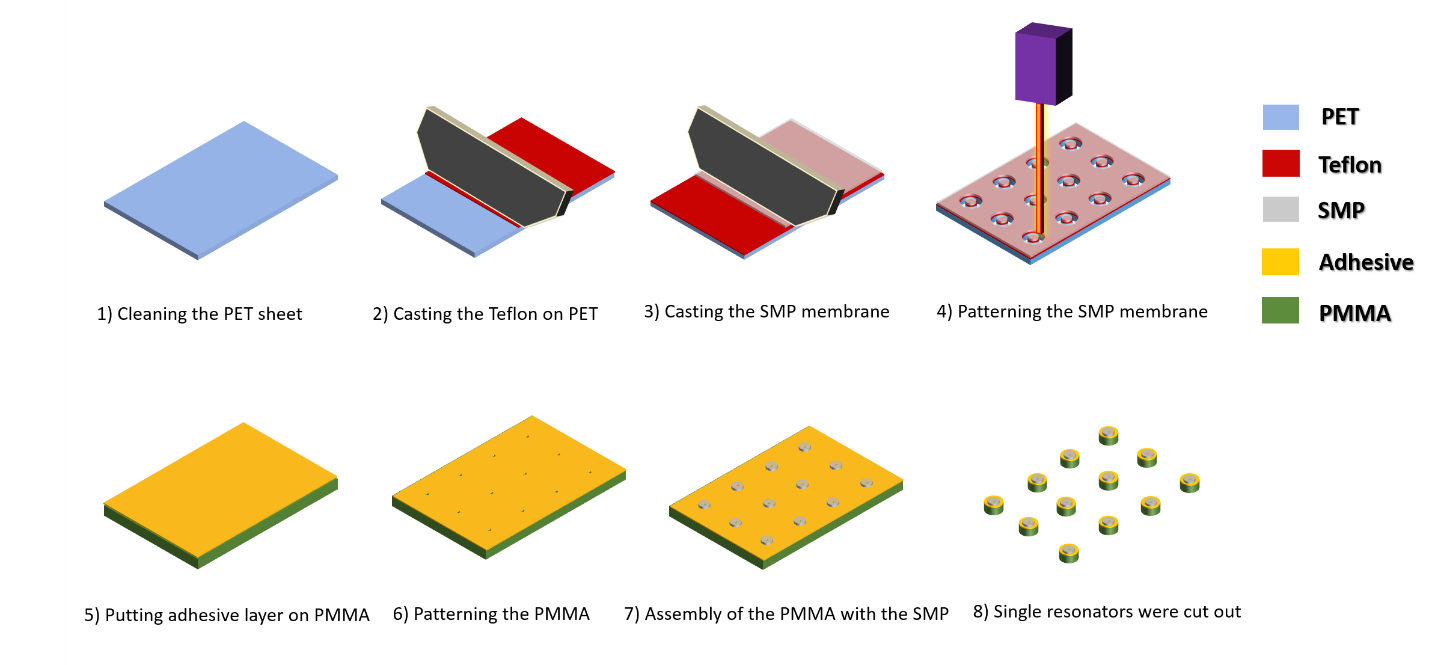


**Supplementary Figure 15:** **Schematic illustration of the fabrication process of the shape memory polymer (SMP) resonators**. The steps for preparing the solution and casting of the SMP is similar to the one used in ref. 36. The resonator thickness is 10 μm.

**Supplementary Notes**

**Supplementary Note 1. Thermal design of the resonator**

A thermal model is essential to estimate the temperature change due to absorbed infrared (IR) radiation on the sensor as well as to assess the thermal time constant, which determines the response time of the sensor and the integration time for the readout. Supplementary Figure 12 shows the cross-section of the shape memory polymer (SMP) resonator with the developed equivalent circuit model.

Supplementary Equation (1) shows the temperature change due to the absorbed IR radiation having power of *P*, where *q* is the heat flux, $\eta$ is the absorption coefficient, $A_{\mathrm{sensor}}$ is the sensor absorption area, $G_{\mathrm{total}}$ is the total thermal conductance (equivalent thermal conductance), $C_{\mathrm{total}}$ is the total thermal capacity and $\omega_{IR}$ is the angular frequency of the modulated IR radiation. As the last part of the Supplementary Equation (1) implies, for DC or very low modulation frequency ($\omega_{\mathrm{IR}}\approx0$), the temperature change directly depends on the thermal conductance${(G}_{\mathrm{total}})$, which is generally the case.

| $\Delta T=\frac{P}{\sqrt{G_{\mathrm{total}}^{2}+{\omega_{\mathrm{IR}}}^{2}C_{\mathrm{total}}^{2}}}=\frac{q\eta A_{\mathrm{sensor}}}{\sqrt{G_{\mathrm{total}}^{2}+{\omega_{\mathrm{IR}}}^{2}C_{\mathrm{total}}^{2}}} \approx\frac{q\eta A_{\mathrm{sensor}}}{G_{\mathrm{total}}}$ | (1) |
| --- | --- |
|  |  |

Supplementary Equation (2) shows the expression for finding the total thermal conductance^1^, $G_{\mathrm{total}}$. $G_{\mathrm{rad}}$ is the radiative thermal conductance, which is originated from the radiative heat exchange between the sensor and the environment. It is the intrinsic thermal conductance of the structure, which emerges as a true limit because all the infrared sensors (cooled/uncooled) are also emitters according to their absorbance based on the Kirchoff’s law^2^. $G_{1}$ and $G_{2}$ is the thermal conductance through the membrane to the substrate, which is related to the geometry of the structure. $G_{3}$ is the thermal conductance between the top and bottom surface of the SMP membrane. The conduction through the air to the substrate is $G_{\mathrm{air}}$, which is significant in atmospheric pressure and negligible in vacuum.

| $G_{\mathrm{total}}=G_{\mathrm{rad}}+\left( \frac{G_{1}G_{2}}{G_{1}+G_{2}} \right)+G_{\mathrm{air}}$ | (2) |
| --- | --- |

Supplementary Equation (3), (4), (5), and (7) provides the thermal conductance for the defined parameters in Supplementary Figure 12 considering a circular membrane. $\sigma$ is the Stefan-Boltzmann constant and $\varepsilon_{\mathrm{SMP}}$ is the emissivity of the SMP, which was assumed same as the absorbance^3^. We utilized these equations for our tested circular membrane Sample #M1 to validate the thermal model with the measurement, where the size of the membrane is determined by the ease of the fabrication. Here are the related parameters: $r$=2.5 mm, $r_{m}$=520 μm, $t$= 10 μm, $t_{\mathrm{air}}$= 1 mm, $k_{\mathrm{SMP}}$=0.18 Wm^-1^K^-1^ (from Supplementary Reference 4) and $k_{\mathrm{air}}$=26.3 x 10^-3^ Wm^-1^K^-1^ (from Supplementary Reference 5).

| $G_{\mathrm{rad}}=\pi r^{2}\sigma\varepsilon_{\mathrm{SMP}}{(T}_{\mathrm{ambient}}^{4}- T_{\mathrm{sensor}}^{4})/(T_{\mathrm{ambient}}-T_{\mathrm{sensor}})$ | (3) |
| --- | --- |
| $G_{1}=k_{\mathrm{SMP}}\frac{2\pi rt}{r}=2\pi k_{\mathrm{SMP}}t$ | (4) |
| $G_{2}=k_{\mathrm{SMP}}\frac{\pi({r_{m}^{2}-r}^{2})}{t}$ | (5) |
| $G_{3}=k_{\mathrm{SMP}}\frac{\pi r^{2}}{t}$ | (6) |
| $G_{\mathrm{air}}=k_{\mathrm{air}}\frac{\pi r^{2}}{t_{\mathrm{air}}}$ | (7) |

$G_{1}$ depends only on the thermal conductivity of the SMP and the thickness of the membrane (Supplementary Equation (4)). For the given geometry in Supplementary Figure 12, $G_{1}$ is very small compared to $G_{2}$ and $G_{3}$, thus, $G_{2}$ and $G_{3}$are negligible in the calculations. Since$G_{3}$ is negligible as compared to $G_{1}$ and $G_{\mathrm{air}}$ (e.g. $G_{3}$ is 3 orders of magnitude higher than$G_{1}$ for *t*=50 μm, which refers to the resistor from the membrane to the substrate), the temperature is same at the top and the bottom surface of the SMP membrane due to an absorbed IR radiation. As it is shown in Supplementary Figure 13.a, $G_{\mathrm{total}}$ is significantly determined by $G_{1}$ in vacuum, $G_{1}$ and $G_{\mathrm{air}}$ in atmospheric pressure. Although $G_{\mathrm{rad}}$ is more than 10 times smaller than $G_{1}$, it has also contribution to the total thermal conductance. For the radius (520 μm) of our sample (Sample #M1), $G_{\mathrm{total}}$ is calculated as 1.4 x 10^-5^ WK^-1^ in vacuum and 3.6 x 10^-5^ WK^-1^ in air. It is very important to note that, the thermal conductance in vacuum and in air is almost same and determined by the thermal conductance of the membrane for smaller radius (<100 μm). It is due to smaller sensor area, which leads to smaller $G_{\mathrm{air}}$ and $G_{\mathrm{rad}}.$ Supplementary Figure 13.b shows the thermal conductance with respect to thickness while keeping the radius fixed (520 μm). Accordingly, the thermal conductance of the membrane and the total thermal conductance (in air and in vacuum) is linearly related with the thickness. Thus, in order to decrease the thermal conductance, one needs to decrease the thickness.

Following the analysis of the total thermal conductance, the total heat capacity of the membrane should also be determined in order to find the thermal time constant ($\tau_{\mathrm{th}}$) of the membrane. The calculation of the total heat capacity of the membrane is expressed in Supplementary Equation (8), where $\rho$ refers to the density of the SMP membrane which was measured as 1100 kgm^-3^. *c* refers to the specific heat capacity, and has values changing from 1200 Jkg^-1^K^-1^ to 1800 Jkg^-1^K^-1^ between 25 °C to 50 °C according to the differential scanning calorimetry (DSC) measurements of SMP^6^.

| $C_{\mathrm{total}}=\left( \pi r^{2}t \right)\rho c$ | (8) |
| --- | --- |

The thermal time constant of the SMP membrane, can be expressed as $\tau_{\mathrm{th}}=\frac{C_{\mathrm{total}}}{G_{\mathrm{total}}}$ . Supplementary Figure 14.a and Supplementary Figure 14.b analyzes the effect of the radius and the thickness of the membrane on the thermal time constant respectively. In order to decrease the time constant, one needs to decrease the radius and/or the thickness of the membrane, while considering the effect of both parameters on NETD via the thermal conductance and the absorber area.

As the star indicators spot out in Supplementary Figure 14, thermal time constant are 335 ms in air and 885 ms in vacuum (at *T*_sub_=30 °C) from the thermal model, where the exponential fit from the measurements reveal time constants of 210 $\pm$ 10 ms and 376$\pm$ 8 ms. The differences in time constants between the experimental data and the thermal model analysis can be attributed to the differences in material properties of the SMP in real life and in the calculations. Although, these time constant values seem high for video applications, it is straightforward to decrease the time constant. For instance, 10 times decrease in the radius (from 520 μm to 52 μm) makes the thermal time constant 100 times lower in vacuum, results in 7 ms according to our model, which is quite sufficient for video applications. In addition, according to our model, this reduction results in almost same time constant in air. This is very important because the thermal conductance through air becomes negligible (very small compared to thermal conductance through the membrane) for these dimensions; which results in the same high performance for atmospheric pressure condition as it is in vacuum (Supplementary Figure 13.a).

We calculate the thermal time constant of the SiNx/SMP resonators by modifying the thermal model slightly, as the geometry changes from a circular membrane to square membrane and as the thickness of the SMP changes from 10 to 15 μm. The calculated thermal time constant from the thermal model is 876 ms for 1 mm x 1 mm and 240 ms for 500 μm x 500 μm sized SiNx/SMP square membranes in vacuum at *T*_sub_ =30 °C, whereas the exponential fit from the measurements in vacuum reveal time constants of 311$\pm$ 1 ms (for 1 mm x 1 mm membrane) and 225 $\pm$ 6 ms (for 500 μm x 500 μm membrane) respectively (See Figure 5.a of the manuscript and Supplementary Figure 9.a). So, the thermal model and experimental results show that the decrease in the size of the membrane decreases the thermal time constant.

Consequently, it is straightforward to adjust the speed of the sensor for video applications and enable the operation of the sensor in atmospheric pressure by maintaining the same performance in vacuum. Decreasing the radius (or the size) of the membrane will decrease the absorption area and degrade the performance. Therefore, some modifications for the dimensions are essential such as decreasing the thickness of the SMP membrane, which results in a decrease in thermal conductance and balances the degradation the performance while still evaluating the thermal time constant to be compatible with video applications.

**Supplementary Note 2 for NETD calculation**

The noise-equivalent temperature difference (NETD) is the most widely used figure of merit for the performance of IR sensor systems. It determines the minimum detectable temperature difference for an IR target (i.e., IR object, the radiation source). In order to find the NETD^7^, one divides the temperature difference of the IR target with the ambient by the signal-to-noise ratio (SNR) of the detection system to obtain the minimum detectable temperature difference for an *SNR* = 1. For resonant IR sensors, the TCF determines the signal level for the SNR calculations. The overall noise of the system is the second parameter that defines the SNR, which can be extracted from Allan deviation ($\sigma_{A}$) measurements. Therefore, $\frac{\sigma_{A}}{TCF}$ gives the minimum detectable temperature for an SNR of 1, which corresponds to the direct temperature sensing as a temperature sensor.

In order to convert this minimum detectable temperature to the IR target (i.e. IR source or object) plane, one needs to consider the temperature change on the sensor that corresponds to the change on the IR target. There are many different parameters that influence the detector to target temperature ratio such as the transmission of the IR optical system (includes atmospheric transmission, transmission of the optical windows and lenses), the collection efficiency of the IR lens system and emissivity of the IR target ($\varepsilon=1$, assuming an ideal blackbody), the absorbance and area of the IR sensor. Supplementary Equation (9) represents the detector to target temperature ratio ($\frac{\Delta T_{D}}{\Delta T_{T}}$), where $\eta$ is the absorbance of the detector, $\tau_{0}$ is the transmittance of the IR optical system through atmosphere and IR optics), $\left( dP/dT \right)_{\lambda1-\lambda2}$ is the radiated power change per temperature change for the IR wavelengths between $\lambda1$=8 µm and $\lambda2$=14 µm per unit area [SI unit: W m^-2^K^-1^], $F_{\#}$ is the f-number of the IR lens system and $G_{\mathrm{total}}$ [SI unit: W m^-1^] is the total thermal conductance of the system^8^:

| $\frac{\Delta T_{D}}{\Delta T_{T}} \approx\frac{\eta\tau_{0}A_{\mathrm{sensor}}\left( dP/dT \right)_{\lambda1-\lambda2}}{4F_{\#}^{2}G_{\mathrm{total}}}$ | (9) |
| --- | --- |

After using Supplementary Equation (9) for our tested sample Sample #M1, we found the $\Delta T_{D}/\Delta T_{T}$ as ~ 0.003, which shows that 1 °C temperature change on the IR target causes 3.3 m°C change on the sensor in vacuum. Similarly, 1 °C temperature change on the IR target causes 1.2 m°C in air. In this calculation, $\tau_{0}$ is 0.7 according to the specifications of the optical window and lenses, $\eta$ is 0.44 according to FTIR measurement, $\left( dP/dT \right)_{\lambda1-\lambda2}$ is 2.62 Wm^-2^K^-1^ for the operation wavelength range^8^, $F_{\#}$ is 2 and $G_{\mathrm{total}}$ was already calculated for vacuum and air.

By optimizing the parameters such as thermal conductance and absorbance, and by using an IR optical system with higher collection efficiency (with low $F_{\#}$), one can increase this detector to target temperature ratio so the sensitivity of the IR sensor. In order to define the sensitivity of the system one needs to find the overall noise of the system. To this aim, many different sources that may contribute to the total noise of the resonant IR detectors should be considered as they are discussed in the following parts.

**Background Fluctuation Noise**

There is a fundamental limit in the thermal isolation of the sensor that is caused by the radiative heat exchange. The temperature change of the background leads to continuing heat exchange between the sensor and its environment through radiation. It is important to express that, all type of IR sensors aim to absorb IR radiation, which also makes them good emitters for the same waveband^3^. Thus, any IR detector (cooled or uncooled) cannot avoid from this radiative heat exchange, which sets the fundamental limit for all IR detectors. Supplementary Equation (10) shows the *NETD_BF_,*  which detector to target temperature ratio was already included to the expression^9^.

| ${NETD}_{\mathrm{BF}}=\frac{8F_{\#}^{2}}{\eta\tau_{0}\left( dP/dT \right)_{\lambda1-\lambda2}}\sqrt{\frac{2k_{B}\sigma_{T}B(T_{D}^{5}+T_{B}^{5})}{A_{\mathrm{sensor}}}}$ | (10) |
| --- | --- |

Some of the parameters in the expression were already defined. Other than these parameters, the ${NETD}_{\mathrm{BF}}$ depends on the $k_{B}$ (Boltzmann’s constant - [SI unit: JK^-1^]), $\sigma_{T}$(Stephan-Boltzmann constant - [SI unit: Wm^-2^K^-4^]), *B* (measurement bandwidth [SI unit: Hz]), $T_{D}$and $T_{B}$ (Detector and background temperature respectively [SI unit: K]).

**Temperature Fluctuation Noise**

In addition to the temporal fluctuations of temperature due to the radiative heat exchange, the thermal conduction through the membrane or the air to the substrate constitutes as another fundamental limitation for all thermal detectors (only uncooled detectors). In order to eliminate the effect of the thermal conductance through the air, most of the thermal detectors typically operate at low pressures (~1e-5 mbar). Since there are temporal fluctuations of temperature at the membrane by means of thermal conduction through the membrane to the substrate, it is called as the temperature fluctuation noise. Supplementary Equation (11) shows the expression of the ${NETD}_{\mathrm{TF}}$ related to the temperature fluctuation noise. All the parameters used in Supplementary Equation (11) were also indicated in the previous subsections.

| ${NETD}_{\mathrm{TF}}=\frac{8F_{\#}^{2}T_{D}\sqrt{k_{B}BG_{\mathrm{total}}}}{\eta\tau_{0}A_{d}\left( dP/dT \right)_{\lambda1-\lambda2}}$ | (11) |
| --- | --- |

**Thermomechanical noise**

Thermomechanical noise is another fundamental noise source for mechanically movable thermal detectors apart from background and temperature fluctuation noise. The vibrational noise originated from the thermal energy ($k_{B}T$) causes the thermomechanical noise through the continuous exchange of the mechanical energy on the sensor. Supplementary Equation (12) shows the expression^10^ for the ${NETD}_{\mathrm{TM}}$, where $E_{c}$ refers to the carrier energy representing the maximum drive energy^11^.

| ${NETD}_{\mathrm{TM}}=\frac{4F_{\#}^{2}G}{\eta\tau_{0}A_{d}\left( dP/dT \right)_{\lambda1-\lambda2}}\frac{1}{TCFw_{0}}\sqrt{\frac{k_{B}TBw_{0}}{E_{c}Q}}$ | (12) |
| --- | --- |
| $E_{c}=M_{\mathrm{eff}}w_{0}^{2}\left\langle x_{c}^{2} \right\rangle$ | (13) |

Supplementary Equation (13) shows the expression of the carrier energy for a circular membrane, where $M_{\mathrm{eff}}$ is the effective mass of the membrane (0.2695m for the flexural mode (0,1) for a circular membrane^12^, where m is the mass of the membrane) and $x_{c}$ is the maximum displacement can be achieved by our circular membranes ($x_{c}$ is accepted as 100 nm).

For the calculation of the ${NETD}_{\mathrm{TM}}$for different thicknesses (at *T*_sub_=30 °C), *w*_0_ is used from the measurements ($w_{0}=2\pi f_{\mathrm{Res}},$ where $f_{\mathrm{Res}}$ was measured as 25.53 kHz for our membrane having a thickness of 10 µm) and then *w*_0_ is modified linearly for the other thicknesses. The Q-factor and TCF obtained from the measurements are Q=34 and 1% K^-1^ respectively and they are accepted same for all thicknesses. The measurement bandwidth (*B*) is taken as 1 Hz to be coherent with the thermal time constant of Sample #M1. The other parameters are already introduced in the calculation of detector to target temperature ratio ($\frac{\Delta T_{D}}{\Delta T_{T}}$).

The explained fundamental noise sources contribute to the total noise of the system independently and determine the sensitivity of our resonant IR sensors. All these noise sources are independent random processes, hence the root mean square value of the noise sources were calculated as the NETD for the whole system as ${NETD}_{\mathrm{TOTAL}}$ in Supplementary Equation (14).

| ${NETD}_{\mathrm{TOTAL}}=\sqrt{{{NETD}_{\mathrm{BF}}}^{2}+{{NETD}_{\mathrm{TF}}}^{2}+{{NETD}_{\mathrm{TM}}}^{2}}$ | (14) |
| --- | --- |

Supplementary Figure 10 shows our calculations for the fundamental NETD noise sources with the total NETD with respect to thickness of the membrane, while keeping the radius of the membrane fixed at 520 μm. Considering the Sample #M1 in vacuum, the calculated total NETD is limited by the thermomechanical (TM) noise to a certain thickness value (~ 20 μm). The reason for the huge TM noise for small thicknesses (<2 μm) are due to the decrease in the resonance frequency with thickness. On the other hand, in atmospheric pressure, the NETD performance, for the thicknesses smaller than 30 μm, slightly degrades as compared to vacuum due to thermal conductance of air.

In the Supplementary Note 1 on the thermal design, we explained how the speed of the sensor can be adjusted for video applications by decreasing the radius of the membrane. In order to evaluate the effect of the thickness on the performance of the IR sensor, the fundamental NETD noise sources with the total NETD were analyzed in regard to the thickness of the membrane (Supplementary Figure 11). In addition, in the case of the radius reduction to 52 μm, the NETD calculations were plotted in vacuum and in air to determine the thickness. As Supplementary Figure 11 shows, the optimum thickness for a membrane with a radius of 52 μm, is around 1.5 μm; which would provide 9 mK NETD for F#2 and 50% absorption in vacuum as well as in air, despite the reduction in the absorber area around 100 times.

All these noise sources influence the frequency stability of our IR resonant sensors. But the thermomechanical noise and the temperature fluctuation noise are the most significant ones for our system, which set a fundamental noise limit for these sensors independent from the read-out noise. Furthermore, the radius reduction at the membrane to achieve high speed for video applications should be accompanied with thickness reduction to attain high performance.

**Supplementary Note 3 for NETD measurements**

The measured NETDs in Figure 4.c of the manuscripts can be compared with a predicted NETD, which is the combination of the measured temperature detection sensitivity with the theoretical value of $\frac{\Delta T_{D}}{\Delta T_{T}}$ , the detector to target temperature ratio (see Supplementary Note 2). In order to calculate the predicted NETD, Supplementary Equation (15) are utilized.

| $NETD=\frac{\Delta T_{D}}{\Delta T_{T}}\frac{\sigma_{A}}{TCF}$ | (15) |
| --- | --- |

Thus, for the predicted NETD, we used the $\frac{\sigma_{A}}{TCF}$measurements from Figure3.c of the manuscript and the computed theoretical $\frac{\Delta T_{D}}{\Delta T_{T}}$using the specifications and assumptions based on the IR optical system.

The best measured NETD is 22 mK (in vacuum, at *T*_sub_ = 30 °C) and 112 mK (in air, *T*_sub_ *=* 25 °C) using an optical system having an f-number of 2 (F#=2). Although these values are consistent with the predicted NETD, the difference between the measured and the predicted NETD increases as the operation temperature increases (Supplementary Figure 7). In our predicted NETD calculations we assumed a fixed absorption and a fixed thermal conductivity for the SMP. These values may be temperature dependent, which could explain part of the difference.

**SUPPLEMENTARY REFERENCES**

1. Jutzi, F., Wicaksono, D. H. B., Pandraud, G., de Rooij, N. & French, P. J. Far-infrared sensor with LPCVD-deposited low-stress Si-rich nitride absorber membrane: Part 2: Thermal property, and sensitivity. *Sens. Actuators Phys.* **152**, 126–138 (2009).

2. Zhao, Y. *et al.* Optomechanical uncooled infrared imaging system: design, microfabrication, and performance. *J. Microelectromechanical Syst.* **11**, 136–146 (2002).

3. Bergman, T. L., Incropera, F. P., DeWitt, D. P. & Lavine, A. S. *Fundamentals of heat and mass transfer*. (John Wiley & Sons, 2011).

4. Azra, C. D. Shape memory polymers with controlled time-dependent shape recovery. (EPFL, 2013).

5. Zhao, Y. Optomechanical uncooled infrared imaging system. (University of California, Berkeley, 2002).

6. Besse, N., Rosset, S., Zarate, J. J. & Shea, H. Flexible active skin: large reconfigurable arrays of individually addressed shape memory polymer actuators. *Adv. Mater. Technol.* **2**, 1700102 (2017).

7. Adiyan, U., Civitçi, F., Ferhanoğlu, O., Torun, H. & Urey, H. A 35-um Pitch IR Thermo-Mechanical MEMS Sensor With AC-Coupled Optical Readout. *IEEE J. Sel. Top. Quantum Electron.* **21**, 87–92 (2015).

8. Toy, M. F., Ferhanoglu, O., Torun, H. & Urey, H. Uncooled infrared thermo-mechanical detector array: Design, fabrication and testing. *Sens. Actuators Phys.* **156**, 88–94 (2009).

9. Datskos, P. G., Lavrik, N. V. & Rajic, S. Performance of uncooled microcantilever thermal detectors. *Rev. Sci. Instrum.* **75**, 1134–1148 (2004).

10. Zhang, X. C., Myers, E. B., Sader, J. E. & Roukes, M. L. Nanomechanical torsional resonators for frequency-shift infrared thermal sensing. *Nano Lett.* **13**, 1528–1534 (2013).

11. Ekinci, K. L., Yang, Y. T. & Roukes, M. L. Ultimate limits to inertial mass sensing based upon nanoelectromechanical systems. *J. Appl. Phys.* **95**, 2682–2689 (2004).

12. Hauer, B. D., Doolin, C., Beach, K. S. D. & Davis, J. P. A general procedure for thermomechanical calibration of nano/micro-mechanical resonators. *Ann. Phys.* **339**, 181–207 (2013).

1. Soft Transducers Laboratory, École Polytechnique Fédérale de Lausanne (EPFL), 2000 Neuchâtel, Switzerland. 2 Advanced NEMS Group, École Polytechnique Fédérale de Lausanne (EPFL), 1015 Lausanne, Switzerland. Correspondence and requests for materials should be addressed to H.S. (email: herbert.shea@epfl.ch) [↑](#footnote-ref-1)
